# Using Molecular Epidemiology to Track *Toxoplasma gondii* from Terrestrial Carnivores to Marine Hosts: Implications for Public Health and Conservation

**DOI:** 10.1371/journal.pntd.0002852

**Published:** 2014-05-29

**Authors:** Elizabeth VanWormer, Melissa A. Miller, Patricia A. Conrad, Michael E. Grigg, Daniel Rejmanek, Tim E. Carpenter, Jonna A. K. Mazet

**Affiliations:** 1 Wildlife Health Center, One Health Institute, School of Veterinary Medicine, University of California, Davis, Davis, California, United States of America; 2 Marine Wildlife Veterinary Care and Research Center, California Department of Fish and Wildlife, Santa Cruz, California, United States of America; 3 Department of Pathology, Microbiology and Immunology, School of Veterinary Medicine, University of California, Davis, Davis, California, United States of America; 4 Laboratory of Parasitic Diseases, National Institutes of Health, National Institute of Allergy and Infectious Diseases (NIAID), Bethesda, Maryland, United States of America; 5 EpiCentre, Massey University, Palmerston North, New Zealand; New York University, United States of America

## Abstract

**Background:**

Environmental transmission of the zoonotic parasite *Toxoplasma gondii*, which is shed only by felids, poses risks to human and animal health in temperate and tropical ecosystems. Atypical *T. gondii* genotypes have been linked to severe disease in people and the threatened population of California sea otters. To investigate land-to-sea parasite transmission, we screened 373 carnivores (feral domestic cats, mountain lions, bobcats, foxes, and coyotes) for *T. gondii* infection and examined the distribution of genotypes in 85 infected animals sampled near the sea otter range.

**Methodology/Principal Findings:**

Nested PCR-RFLP analyses and direct DNA sequencing at six independent polymorphic genetic loci (B1, SAG1, SAG3, GRA6, L358, and Apico) were used to characterize *T. gondii* strains in infected animals. Strains consistent with Type X, a novel genotype previously identified in over 70% of infected sea otters and four terrestrial wild carnivores along the California coast, were detected in all sampled species, including domestic cats. However, odds of Type X infection were 14 times higher (95% CI: 1.3–148.6) for wild felids than feral domestic cats. Type X infection was also linked to undeveloped lands (OR = 22, 95% CI: 2.3–250.7). A spatial cluster of terrestrial Type II infection (P = 0.04) was identified in developed lands bordering an area of increased risk for sea otter Type II infection. Two spatial clusters of animals infected with strains consistent with Type X (P≤0.01) were detected in less developed landscapes.

**Conclusions:**

Differences in *T. gondii* genotype prevalence among domestic and wild felids, as well as the spatial distribution of genotypes, suggest co-existing domestic and wild *T. gondii* transmission cycles that likely overlap at the interface of developed and undeveloped lands. Anthropogenic development driving contact between these cycles may increase atypical *T. gondii* genotypes in domestic cats and facilitate transmission of potentially more pathogenic genotypes to humans, domestic animals, and wildlife.

## Author Summary

*Toxoplasma gondii*, a global parasite shed by domestic and wild felids, can cause severe disease in people and animals. In coastal California, USA, many sea otters have died due to *T. gondii*. Because *T. gondii* is shed by felids on land, otter infection suggests that this extremely hardy parasite is transported in freshwater runoff to aquatic environments, where animals and humans can become exposed. Molecular characterization of *T. gondii* strains infecting terrestrial and marine hosts can provide clues about parasite transmission cycles and sources of otter infection. By testing 373 and characterizing *T. gondii* infection in 85 terrestrial carnivores (domestic cats and wild carnivores) sharing the California coast, we found that Type X, the type previously identified in over 70% of infected sea otters tested, was more common in wild felids than domestic cats. However, discovery of Type X in domestic cats in this region suggests that they may play an important role in marine infection, as their populations are larger than those of wild felids. Differences in types of *T. gondii* among carnivores and in urban and agricultural vs. undeveloped areas suggest that there are separate, but overlapping domestic and wild cycles of *T. gondii* transmission in coastal California.

## Introduction

As human populations expand and alter global habitats, increased contact among people, domestic animals, and wildlife can lead to the emergence or re-emergence of diseases that threaten public and animal health [1]–[3]. Originally described in terrestrial environments, the zoonotic protozoan parasite, *Toxoplasma gondii,* is emerging as an important pathogen in aquatic systems. *Toxoplasma gondii* has been linked to widespread marine mammal infection and severe water-borne disease outbreaks in humans around the world [4]. The impact of *T. gondii* has been particularly significant in the threatened southern sea otter (*Enhydra lutris nereis*) population along California's central coast, where it has caused illness and death of otters of prime reproductive age [5], [6]. In addition to playing a vital role in maintaining near-shore kelp forest ecosystems, sea otters serve as important sentinels for identifying disease threats, like *T. gondii*, to other marine species and humans sharing the coastal environment [6], [7].

Warm-blooded animals and humans typically become infected with *T. gondii* by ingesting oocysts in contaminated water or food, by eating an intermediate host with parasite cysts in its tissues, or via vertical (e.g. transplacental or transmammary) transmission [8]. Oocysts, the extremely hardy, free-living stage of *T. gondii* [9]–[11], play a key role in terrestrial and aquatic environmental transmission [12], [13]. Epidemiologic association of sea otter *T. gondii* infection with diet, as well as laboratory and field evidence that marine invertebrates can filter and concentrate oocysts or collect oocysts while feeding on kelp, support the high likelihood of otter infection through oocyst-contaminated prey [14]–[18]. Because humans consume many of the same marine invertebrates, such as raw oysters, coastal contamination with *T. gondii* poses a public health risk [19]. As domestic and wild felids are the only known hosts capable of shedding *T. gondii* oocysts, high levels of marine mammal infection suggest land-to-sea transmission. Oocysts shed in felid feces become infective in the environment and can accumulate and survive for over a year in soil, freshwater, and seawater under a range of ambient conditions [20]–[22]. Environmental persistence increases the potential for oocysts to be transported in freshwater runoff to aquatic systems where sea otters, people, and other animals can become exposed. A terrestrial source of *T. gondii* is also supported by research linking increased sea otter *T. gondii* infection to coastal freshwater runoff [23]. Oocysts shed in the feces of pet and feral domestic cats, as well as wild felids, can serve as the source of sea otter and human infection, but contributions of these different felids to environmental oocyst contamination are not clearly defined.

Molecular epidemiology offers a unique approach to trace pathogens from their sources to hosts in diverse environments [24]–[26]. Previous work has linked *T. gondii* genetics to infection patterns at both host population and landscape levels [27]–[29]. There is only one species of *Toxoplasma*, but different genotypes have been described based on the alleles present at multiple loci in *T. gondii* isolates [28], [30], [31]. Early population genetic analyses on *T. gondii*, primarily on isolates collected from domestic animals and humans in North America and Europe, identified three predominant, archetypal clonal lineages; Types I, II, and III [32]. However, in Central and South America, diverse atypical genotypes were later found infecting wildlife, domestic animals, and people [31], [33]–[35]. Archetypal genotypes appear to dominate in humans and domestic animals in North America [31], while atypical genotypes are more prevalent in wildlife [27], [36]–[38]. Over 70% of *T. gondii* isolates from California sea otters sampled between 1998 and 2004 were identified as a novel, atypical genotype, named Type X [6], [39]. Type X (also designated Type 12, Haplogroup 12; HG12, and ToxoDB Genotype #5) and other closely related atypical genotypes commonly infect diverse wildlife species in the continental United States [27], [31]. In coastal California, *T. gondii* strains consistent with Type X were previously found in three wild felids and one fox, but only archetypal genotypes were detected in the five domestic cats tested [16]. Separate, co-existing domestic and wild (sylvatic) cycles of *T. gondii* transmission may explain the distribution of archetypal and atypical genotypes in different hosts [40], [41]. In French Guiana, these cycles have been linked to human influences on the landscape, with archetypal genotypes associated with domestic animals in anthropized or developed areas and atypical genotypes predominant in people and wildlife from undeveloped tropical rainforest [29], [42].

To evaluate terrestrial sources of California sea otter *T. gondii* infection, and by proxy, potential sources of human exposure, we focused on two central research questions. 1) Do both domestic and wild felids contribute to the environmental transmission of Type X oocysts? 2) How does human land use influence terrestrial and land-to-sea transmission of archetypal and atypical *T. gondii?* Central coastal California offers an ideal environment to investigate spatial and species-based patterns of *T. gondii* infection. Sympatric wild and domestic felid populations share the landscape bordering the sea otter range, a mosaic of developed urban, rural, and agricultural lands and “wild” areas with minimal or no development. We aimed to identify *T. gondii* genotypes infecting a large sample of animals from sympatric populations of domestic cats, wild felids, and wild canids as well as risk factors for Type X infection and spatial clusters of archetypal and atypical *T. gondii* infection in the coastal environment. Although they do not contribute *T. gondii* oocysts to the environment, we included wild canids as sentinels of the genotypes circulating in the coastal lands within their limited home ranges. We use the types of *T. gondii*, risk factors, and spatial clusters identified in our analyses to explore local terrestrial cycles of *T. gondii* transmission and implications for human and animal health in temperate and tropical landscapes.

## Methods

### Sample collection from 373 terrestrial carnivores

Feral domestic cats (*Felis catus*), bobcats (*Lynx rufus*), mountain lions (*Puma concolor*), red foxes (*Vulpes vulpes*), grey foxes (*Urocyon cinereoargenteus)*, and coyotes (*Canis latrans*) were opportunistically collected within 100 kilometers of Moss Landing (36°48'15.28" N, 121°47'13.09" W) and Morro Bay (35°22'16.83" N, 120°51'25.00" W), California, USA, from 2006 through 2009. These locations border marine sites previously identified as areas of increased risk for sea otter infection with *T. gondii* [5], [6], [17], [23], [43].

No animals were euthanized for the purpose of this study, and all animals were sampled through collaborations with regional animal shelters and state and federal wildlife protection programs. Carnivores, which were humanely euthanized or found dead (additional detail in Table S1), were submitted to the California Department of Fish and Wildlife for postmortem examination. The majority (90%) of the free-ranging, unowned feral domestic cats were euthanized by animal shelter population control programs. These cats were identified as feral by study area residents who trapped and submitted them to the shelters or through behavioral testing conducted by trained shelter staff. Additional feral domestic cats and red foxes were trapped and euthanized as part of a conservation program to remove invasive predators from endangered shorebird habitat. Grey foxes were largely killed by cars (42%) or euthanized by wildlife rehabilitation centers (47%). The majority of bobcats (85%) and coyotes (88%) died due to trauma (predominantly caused by vehicles). Most mountain lions (93%) were killed by motor vehicles or shot by wildlife wardens due to predation on domestic animals or due to public safety concerns. Based on postmortem examination, cause of death was not attributed to *T. gondii* in any of the sampled carnivores. Demographic data (species, age, sex, and reported location of death or capture prior to submission to shelter or rehabilitation facility) were recorded during postmortem examination.

Brain and tongue tissue samples collected aseptically during postmortem examination were stored at −80°C prior to molecular testing. Aliquots of freshly collected brain tissue were stored in antibiotic saline at 4°C for parasite culture. To culture *T. gondii* tachyzoites, brain homogenate was inoculated onto uninfected cell culture monolayers as described previously [44]. Cultures were monitored for 30 days, and discarded if no parasite growth was observed. For cultures with parasite growth, tachyzoite-infected cells were harvested, and cell pellets were stored at −80°C prior to molecular testing.

### Multi-locus PCR-RFLP and sequence analysis

We used established nested PCR analyses, restriction fragment length polymorphism (RFLP) genotyping, and direct DNA sequencing at six polymorphic genetic loci (B1, SAG1, SAG3, GRA6, L358, and Apico) to characterize the genotype(s) of *T. gondii* in infected hosts [16], [39]. Although RFLP genotyping approaches have been developed to assess alleles at 11 loci, these methods have primarily been applied to *T. gondii* DNA extracted from isolates rather than tissue samples [30], [45]. Due to the low burden of *T. gondii* bradyzoite cysts in many naturally infected carnivores, genotyping DNA extracted directly from tissues necessarily required us to focus on only a few, highly sensitive genetic loci [16]. However, performing DNA sequence analysis in addition to RFLP genotyping in the present study enhanced our ability to assess variation at target loci. For example, GRA6 sequences differentiate Type II and Type X alleles, which share a common RFLP pattern at this locus. Additionally, choosing loci that were used to characterize genotypes in many of the tested sea otters [6], [39], allowed us to make more consistent terrestrial to marine comparisons.

For each carnivore, brain and tongue samples or culture-derived infected cell pellets were screened for *T. gondii* infection using nested primers targeting the 35 copy B1 locus [46] under previously described thermocyler conditions [47]. As *T. gondii* cysts in tissues of naturally infected animals can be non-uniformly distributed [16], three separate tissue aliquots (one brain, one tongue, and one randomly selected brain or tongue) were tested for each terrestrial carnivore before classifying the animal as negative. Samples that were PCR-positive for *T. gondii* on B1 screening were additionally analyzed at single copy loci with nested SAG1, SAG3, GRA6, L358, and Apico primers [45], [48] using previously described thermocycler conditions [39].

DNA was extracted from 25 milligrams (mg) of cryopreserved tissue or tachyzoite-infected culture cell pellet using DNeasy extraction kits (Qiagen, Valencia, California, USA). The manufacturer's instructions were followed with one modification: AE buffer diluted 1∶10 and heated to 95°C was used in the final elution step. For all target genes, external primer 50 µL PCR reactions included 37.7 µL of DNase- and RNase-free, distilled water; 5 µL of PCR buffer (10X buffer containing 15 millimolar (mM) MgCl_2_); 4 µL of 2.5 mM dNTP mixture; 0.5 µL each of 50 micromolar (µM) forward and reverse primers; 1.5 units Taq Polymerase; and 2 µL of the DNA template. One µL of external primer amplification product was used in the nested reaction. Positive controls (culture-derived and characterized *T. gondii* DNA) and negative controls (reagents only) were included in each round of PCR. Nested amplification products were separated electrophoretically on a 2% agarose gel stained with ethidium bromide, and viewed under UV light. Products consistent in size with *T. gondii*-positive controls were digested with gene-specific restriction enzymes [39], [45] at 37°C for one hour, and banding patterns were visualized under UV light following gel electrophoresis. DNA from PCR products was prepared with QIAquick gel extraction kits (Qiagen) and ExoSAP-IT (Affymetrix, Santa Clara, California, USA) and sequenced at the Division of Biological Sciences DNA sequencing facility (University of California, Davis, USA).

Distinctive RFLP banding patterns and DNA sequences were used to determine the alleles present at target loci [30], [39]. RFLP patterns were used to identify Type I, Type II or III, and Type X alleles at the B1 locus. For B1 samples with atypical RFLP banding patterns and all alleles at the single copy loci, nucleotide sequences of *T. gondii* DNA were aligned and compared to consensus archetypal and Type X sequences using a Clustal W alignment algorithm in MEGA v. 5.05 [49]. Characteristic single nucleotide polymorphisms at established nucleotide positions [39], [46] were evaluated to determine the type of the allele present at each locus. Samples with sequences identical to established archetypal or Type X sequences for a particular locus were classified as having a Type I, II, III, or X allele. Type A, a genotype that has been reported in sea otters and terrestrial wildlife, differs from Type X by a single nucleotide mutation at the GRA6 locus [27], [50]. In order to effectively compare results from this study to previous molecular research on *T. gondii* in California hosts, which did not distinguish between Type X and Type A [6], [16], [39], we consider “Type X” infection to represent both genotypes. For sequences with novel nucleotide polymorphisms at a target locus, the number of mutations to achieve the sample sequence relative to archetypal and Type X consensus sequences was considered. Sequences containing one or two nucleotide mutations compared to related archetypal or Type X consensus sequences were classified as genetically drifted alleles from the closest parent sequence. When more than two mutations were present or sequences were equally distant from established archetypal and Type X sequences based on numbers of nucleotide substitutions, alleles were classified as unique. The inheritance pattern of the alleles across the six independent, polymorphic loci was used to classify the type of *T. gondii* amplified from each infected carnivore.

### Statistical analyses

Associations between *T. gondii* genotype and demographic, temporal, and environmental variables were evaluated using logistic regression. Risk factors for infection with strains of *T. gondii* consistent with Type X versus strains with archetypal alleles or atypical allele mixtures were assessed in carnivores with *T. gondii* DNA amplified at the B1 locus (Model 1, n = 85), as well as a more conservative subset of animals with alleles characterized at two or more loci (Model 2, n = 59). Risk factors evaluated included carnivore group (feral domestic cat, wild felid, or wild canid), age class (juvenile or adult), sex, and season (wet or dry) and year of sampling. As *T. gondii* DNA was only amplified from one coyote, foxes and coyotes were grouped as “wild canids”. Similarly, due to limited sample sizes, mountain lions and bobcats were combined as “wild felids”. These intermediate host (wild canid) and definitive host (wild felid) groupings are epidemiologically reasonable as the pooled animals likely share similar *T. gondii* exposure histories. Although bobcats and mountain lions commonly consume different wild prey species [51]–[53], previous studies in central coastal California found similar seroprevalences of infection and genotypes of *T. gondii* among these species [16], [54]. To classify the season in which each animal was sampled, we used local daily rainfall data from the closest California Irrigation Management Information System (CIMIS) gauge station [55]. Animals collected on dates between the first and last precipitation events recorded from October to May, the time period with the majority of annual rainfall in the coastal study area, were considered “wet season” samples.

Predominant land use (developed vs. undeveloped) within 5 km of the carnivore sampling location was also evaluated as a risk factor for Type X infection. For each carnivore, the street address or a detailed description of the location where the animal was trapped or found dead was recorded. These sampling locations were manually geocoded using Google Earth version 5.2 (Google Inc, Mountain View, California, USA) and mapped on publicly available California land use layers [56], [57] using ArcGIS v. 10 (ESRI, Redlands, California, USA). We used categories in the land use layers to identify developed and undeveloped areas of the coastal landscape. Developed areas included urban and rural lands with residential, commercial, industrial, and agricultural (row crops, hay pastures, orchards, and vineyards) development. Undeveloped areas had little to no human development and included forests, woodlands, grasslands, shrublands, and wetlands. Predominant land use at each carnivore sampling location was determined by buffering the sampling point by 5 km and assessing the proportion of developed vs. undeveloped habitat within the buffer. Two bobcats and four feral domestic cats were excluded from land use analyses due to lack of spatial data.

Risk factors associated with *T. gondii* genotype in univariable logistic regression models (P ≤ 0.20) were included in multivariable models. Potential confounders and interaction variables were evaluated and controlled for in the models as necessary. After incorporating biologically and statistically significant variables (P ≤ 0.05), Akaike's Information Criterion (AIC) was used to select a parsimonious multivariable model for each dataset. Final model fit was evaluated through graphical residual diagnostics. Adjusted odds ratios and respective 95% confidence intervals were estimated to evaluate the magnitude of the association between each variable and *T. gondii* genotype. Although most carnivores were collected from unique locations, there were nine sites where two animals were sampled in the Model 1 data. Model 2 data included five sites with two animals sampled. To account for potential unmeasured correlation among animals sampled at the same site, mixed effects logistic regression models with sampling location as a cluster variable were evaluated. Tests of within location correlation conducted with 5000 bootstrap replicates for each mixed effects model were non-significant (P > 0.05), indicating that conventional logistic regression models were adequate. Statistical tests were performed in R [58] with mixed effects model parameters estimated using the glmmML package [59].

Geographical clustering of *T. gondii* strains consistent with Type X and Type II was evaluated among infected carnivores (animals with sampling location data and alleles characterized for at least two loci) using a Bernoulli model elliptical scanning window, with a medium non-compactness penalty in SatScan v. 9.0 [60], [61]. The default maximum spatial cluster size of 50% of the population at risk was used, and overlapping clusters were not permitted. Wild and domestic carnivores are not randomly distributed in the coastal landscape, and Type X and archetypal genotypes have been more frequently reported in wildlife and domestic cats, respectively. Therefore, the analyses were adjusted for type of animal sampled in order to identify geographic clusters of strains with Type II or X alleles rather than spatial differences in carnivore sampling [62]. A significance level of α = 0.05 was used for all spatial and non-spatial statistical analyses.

## Results

### Types of *T. gondii* infecting domestic and wild carnivores

**Figure 1:**
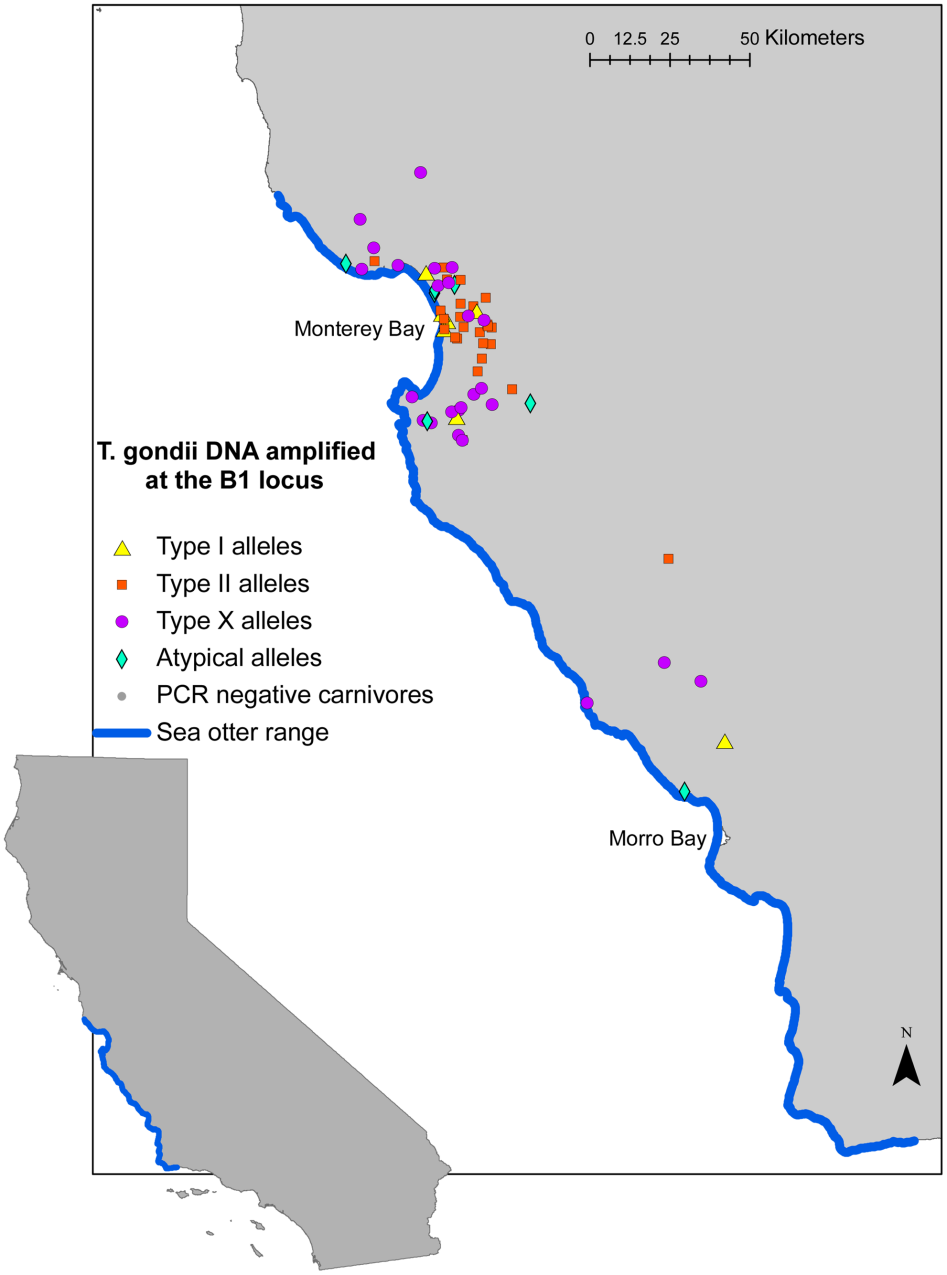
Distribution of *Toxoplasma gondii­*-infected carnivores with alleles characterized at the B1 locus in coastal California. *Toxoplasma gondii* DNA was detected in 85 of 373 terrestrial carnivores (mountain lions, bobcats, foxes, coyotes, and feral domestic cats) sampled along the sea otter range in coastal California, USA, 2006–2009. Alleles present at the B1 locus were identified following nested PCR amplification of DNA extracted from tissue and parasite culture samples, restriction fragment length polymorphism (RFLP) genotyping, and direct sequencing.

**Figure 2:**
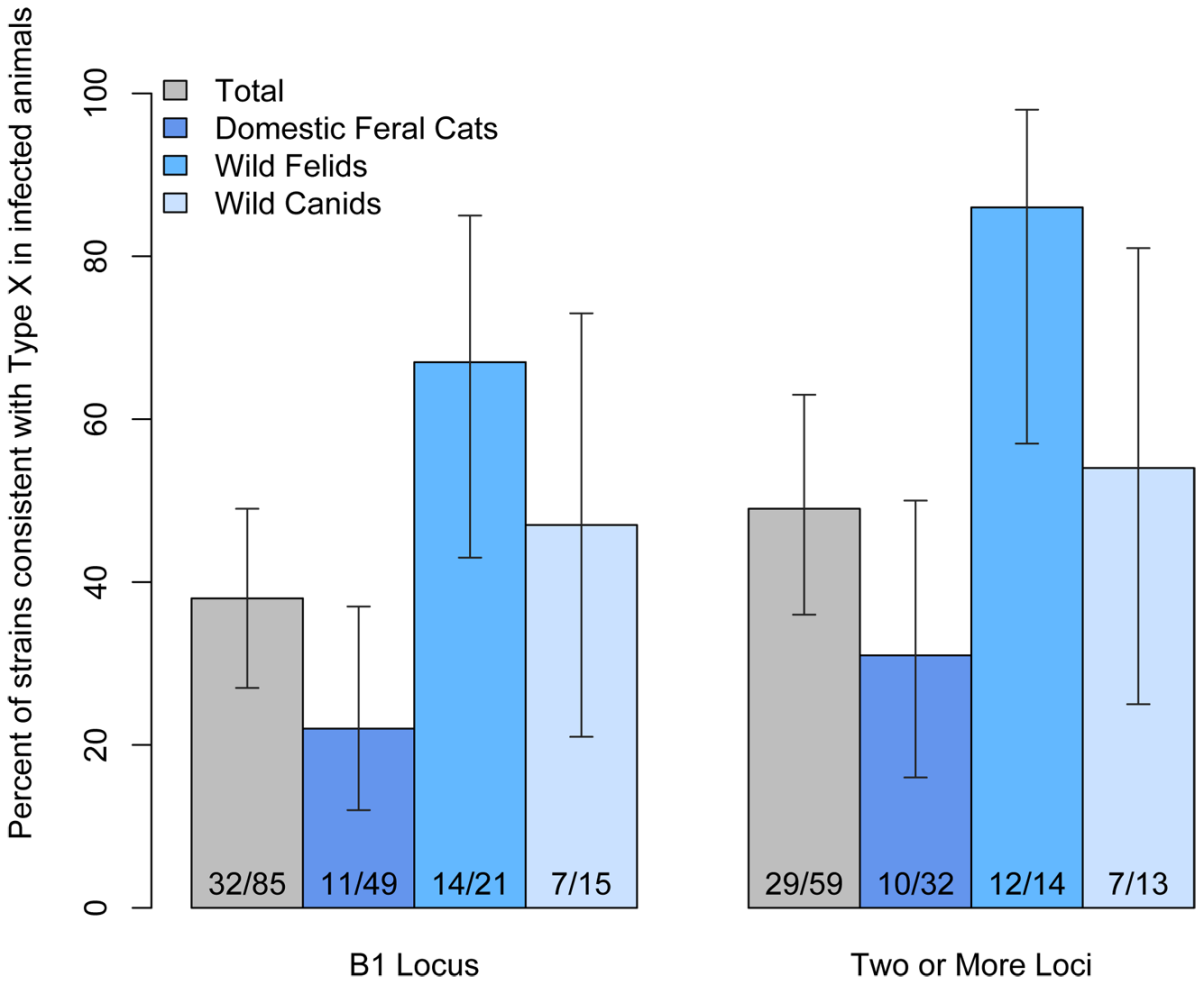
Prevalence of Type X alleles and strains consistent with Type X in *Toxoplasma gondii*-infected carnivores. Type X alleles at the B1 locus were more prevalent in wild felids (mountain lions and bobcats) than feral domestic cats. Strains consistent with Type X remained more prevalent in wild felids within a more conservative subset of carnivores with alleles characterized at B1 and at least one additional locus (SAG1, SAG3, GRA6, L358, and/or Apico). Carnivores were sampled along the California sea otter range, 2006–2009. Error bars represent 95% exact confidence intervals.

From 2006 through 2009, 373 terrestrial carnivores were sampled along the central California coast. *Toxoplasma gondii* DNA was amplified at the B1 locus from 85 (23%) of the sampled animals; 49 (30%) of 166 feral domestic cats, 10 (14%) of 73 mountain lions, 11 (41%) of 27 bobcats, 14 (17%) of 81 foxes, and one (4%) of 26 coyotes (Figure 1). Parasite DNA was amplified from culture-derived, tachyzoite-infected cell pellets (culture) as well as brain and tongue tissue samples (Table 1). Archetypal and atypical alleles of *T. gondii* were detected, with Type X alleles identified at the B1 locus in 32 (38%) of 85 *T. gondii-*infected animals. At B1, Type X alleles were more prevalent in sampled mountain lions and bobcats than in feral domestic cats (Figure 2). Strains consistent with Type X remained more common in wild felids when the dataset was restricted to animals with *T. gondii* alleles identified at two or more loci. In addition to B1 Type X and archetypal alleles, unique atypical alleles were identified in 10 animals based on nucleotide sequence polymorphisms at established positions (Table S2). Genotype characterization of *T. gondii* from the majority of animals with unique alleles at the B1 locus was limited by lack of amplification for single copy loci. A fox and a feral cat were infected with strains of *T. gondii* with an allele pattern consistent with Type II isolates across loci, but each possessed a unique, non-Type II single nucleotide polymorphism (SNP) at SAG3 (Table S3), suggesting low levels of genetic drift. Additionally, one feral cat (FC 1; Table 1) was co-infected with two *T. gondii* strains, one with Type I alleles and one with Type II alleles, which were amplified from tongue and brain tissue, respectively.

**Table 1:** *Toxoplasma gondii* alleles detected in 85 domestic and wild terrestrial carnivores from the central California coast.

Carnivore Species^a^ [ID number (s)]	Sample type	B1 allele	SAG1 allele	SAG3 allele	GRA6 allele	L358 allele	Apico allele	Multi-locus genotype^b^	Land Use^c^
Type I Reference		I	I	I	I	I	I	**I**	
Type II Reference		II or III	II or III	II or X	II	II	II	**II**	
Type II Reference		II or III	II or III	II or X	II	II	I	**II**	
Type III Reference		II or III	II or III	III	III	III	III	**III**	
Type X Reference		X	X	II or X	X	I	I	**X**	
FC [1]	Tongue	I	- d	-	-	-	I	**I**	**D**
Bobcat [1]	Tongue	I	-	-	-	-	-	**-**	**D**
ML [1]	Brain	I	-	-	-	-	-	**-**	**D**
FC [2], [3]	Brain	I	-	-	-	-	-	**-**	**D**
Fox [1]	Brain	I	-	-	-	-	-	**-**	**U**
FC [4]-[11], Fox [2]	Culture	II or III	II or III	II or X	II	II	II	**II**	**D**
FC [12], Fox [3]	Culture	II or III	II or III	II or X	II	II	II	**II**	**U**
FC [13]	Culture	II or III	II or III	II-drift _1_	II	II	I	**II**	**D**
FC [1], [14]–[16], Fox [4]	Culture	II or III	II or III	II or X	II	II	I	**II**	**D**
Bobcat [2]	Tongue	II or III	-	II or X	II	-	I	**II**	**NA**
FC [17]	Tongue	II or III	II or III	II or X	II	-	-	**II**	**D**
Bobcat [3]	Tongue	II or III	-	II or X	-	-	-	**II**	**D**
Fox [5]	Brain	II or III	-	II-drift _2_	-	-	-	**II**	**D**
FC [18]-[22]	Tongue	II or III	-	-	II	-	-	**II**	**D**
FC [23]-[28]	Tongue	II or III	-	-	-	-	-	**-**	**D**
Fox [6]	Tongue	II or III	-	-	-	-	-	**-**	**U**
Bobcat [4]	Brain	X	X	II or X	X	I	I	**X**	**NA**
Fox [7], ML [2]	Brain	X	X	II or X	X	I	I	**X**	**U**
FC [29]	Tongue	X	X	II or X	X	-	I	**X**	**U**
Fox [8], [9]	Tongue	X	X	-	X	-	-	**X**	**U**
Bobcat [5], ML [3]	Tongue	X	-	-	X	-	I	**X**	**U**
FC [30]-[33]	Tongue	X	X	-	X	-	-	**X**	**D**
FC [34], [35]	Tongue	X	-	-	X	-	-	**X**	**NA**
FC [36]	Tongue	X	-	-	X	-	-	**X**	**D**
Fox [10], ML [4]	Tongue	X	-	-	X	-	-	**X**	**U**
Bobcat [6]	Tongue	X	-	-	X	-	-	**X**	**U**
ML [5]	Brain	X	-	-	X	-	-	**X**	**D**
Fox [11], [12], ML [6]	Brain	X	-	-	X	-	-	**X**	**U**
Bobcat [7], Coyote [1]	Brain	X	-	-	X	-	-	**X**	**U**
Bobcat [8]	Tongue	X	-	II or X	-	-	-	**X**	**D**
Bobcat [9], FC [37]	Tongue	X	-	II or X	-	-	-	**X**	**U**
FC [38], ML [7]	Brain	X	-	II or X	-	-	-	**X**	**D**
ML [8], [9]	Brain	X	-	-	-	-	-	**-**	**D**
FC [39], [40]	Tongue	Unique _1_	-	-	-	-	-	**-**	**NA**
FC [41], [42]	Tongue	Unique _1_	-	-	-	-	-	**-**	**D**
Bobcat [10], [11],FC[43]	Tongue	Unique _1_	-	-	-	-	-	**-**	**U**
FC [44], [45]	Brain	Unique _1_	-	-	-	-	-	**-**	**D**
Fox [13]	Tongue	Unique _2_	X	II or X	X	-	**I**	**Atypical**	**D**
Fox [14]	Brain	Unique _2_	II or III	II or X	-	-	**-**	**Atypical**	**U**
FC [46], [47], ML [10]	Tongue	Unique _2_	-	-	-	-	**-**	**-**	**D**
FC [48]	Tongue	I	-	II or X	-	-	**I**	**Atypical**	**D**
FC [49]	Tongue	X	II or III	-	II	-	**I**	**Atypical**	**D**

a FC  =  Feral cat: free-ranging, unowned domestic cats. ML  =  Mountain lion: wild felids of the species *Puma concolor*, also commonly called cougars or pumas.

b Multi-locus genotypes refer to strains consistent with Type I, II, X or Atypical strains based on the alleles at six target loci determined by concatenated sequence and restriction fragment length polymorphism (RFLP) genotyping data. *Toxoplasma gondii DNA* was amplified from postmortem brain or tongue tissue samples or from tachyzoite-infected cell cultures of brain tissue by nested PCR analyses. Genotypes consistent with Type I, II, or X based on fewer than six alleles may be atypical genotypes.

c Predominant land use was characterized for each carnivore based on sampling location (animals sampled 2006–2009). D  =  predominantly developed urban, rural, and agricultural lands, U  =  predominantly undeveloped lands including forests, woodlands, grasslands, shrublands, and wetlands, NA  =  no spatial data available.

d (-)  =  PCR was performed, but *T. gondii* DNA was not amplified.

All animals with *T. gondii* DNA amplified at six loci were identified as infected with Type II (n = 17) or Type X (n = 3) genotypes (Table 1). Characterizing the allele present at the L358 locus allowed us to distinguish between Type II and a Type 12 strain closely related to Type X [27]. One feral cat (FC 29) with alleles determined at five loci was also infected with a *T. gondii* strain consistent with Type X. Four animals (two feral cats and two foxes) infected with strains with a mixture of alleles not consistent with archetypal strains or Type X were classified as atypical. The remainder of the *T. gondii* infected carnivores had DNA amplified at B1 and at least one additional locus (n = 36) or the B1 locus alone (n = 25). While *T. gondii* genotype cannot be fully characterized based on the alleles at one or two loci, we chose loci (B1, SAG1, GRA6) with distinct Type X RFLP patterns or sequence differences to help differentiate between Type X and archetypal strains. It is possible that some of the Type X and Type II strains identified based on fewer loci are actually atypical genotypes, but the likelihood that they were misclassified as Type X instead of archetypal is low. For the remaining results and the discussion, “Type X” refers to *T. gondii* strains with alleles consistent with Type X and “Type II” refers to *T. gondii* strains with alleles consistent with Type II.

### Risk factors for infection with Type X *T. gondii*

Demographic and environmental risk factors were identified for infection with *T. gondii* strains consistent with Type X. When all carnivores with alleles characterized at the B1 locus were included in a multivariable logistic regression model (Model 1), carnivore type was significantly associated with Type X infection (Table 2). The odds of infection with Type X *T. gondii* were almost five times higher in wild felids (bobcats and mountain lions) than in feral domestic cats. The odds of infection with strains consistent with Type X increased to 13.7 times higher in wild felids than feral domestic cats when a more conservative subset of carnivores, those with alleles identified for at least two loci, was examined (Model 2, Table 2). The odds of Type X *T. gondii* infection in wild canids did not differ significantly from domestic cats in either model. Adjusting for carnivore group in multivariate models 1 and 2, land use was a significant risk factor for Type X infection. Carnivores living in predominantly undeveloped lands were six (Model 1) to 22 (Model 2) times more likely to be infected with *T. gondii* strains consistent with Type X. All foxes infected by *T. gondii* strains consistent with Type X (n = 6) lived in predominantly undeveloped lands near sampled mountain lions and bobcats with Type X strains. The majority of foxes with archetypal *T. gondii* infection (3 of 5) lived in urban and agricultural areas near domestic cats infected with these genotypes. Although Type X infection was more likely in feral domestic cats that lived in or closer to undeveloped lands, Type X-infected feral cats were also identified in heavily developed urban and agricultural areas. Season and year of sampling and age were not associated with Type X infection. No significant interactions among variables were identified.

**Table 2:** Risk factors for infection with *Toxoplasma gondii* strains consistent with Type X in domestic and wild terrestrial carnivores.

Model Number^a^: dataset (No. of carnivores included)^b^	Risk Factor (reference category)	Sample size	Adjusted Odds Ratio	95% Confidence Interval	P-value
1: B1 allele type^c^	Carnivore group				
(n = 85)	(Feral cat)	49	1.0	-	-
	Wild felid	21	4.9	(1.3–18.5)	0.02*^d^
	Wild canid	15	1.1	(0.2–5.5)	0.90
	Predominant land use				
	(Developed)	54	1.0	-	-
	Undeveloped	25	6.0	(1.6–23.0)	<0.01*
2: Multi-locus genotypes^e^	Carnivore group				
(n = 59)	(Feral cat)	32	1.0	-	-
	Wild felid	14	13.7	(1.3–146.8)	0.03*
	Wild canid	13	0.5	(0.1–4.9)	0.54
	Predominant land use				
	(Developed)	35	1.0	-	-
	Undeveloped	20	21.9	(2.3–250.7)	<0.01*

a Risk factors were identified using multivariable logistic regression models.

b Carnivores were sampled in central coastal California from 2006–2009.

c *Toxoplasma gondii* type classified based on the allele present at the B1 locus. Spatial data were missing for 6 of the 85 carnivores in this model.

d *  =  statistically significant risk factor for Type X infection, α = 0.05.

e *Toxoplasma gondii* genotypes classified based on the allele present at the B1 locus and sequence data for at least one single copy locus. Carnivores with RFLP data only or sequence data for only B1 were excluded. Spatial data were missing for 4 of the 59 carnivores in this model.

### Geographic distribution of archetypal and atypical *T. gondii* in central coastal California

**Figure 3:**
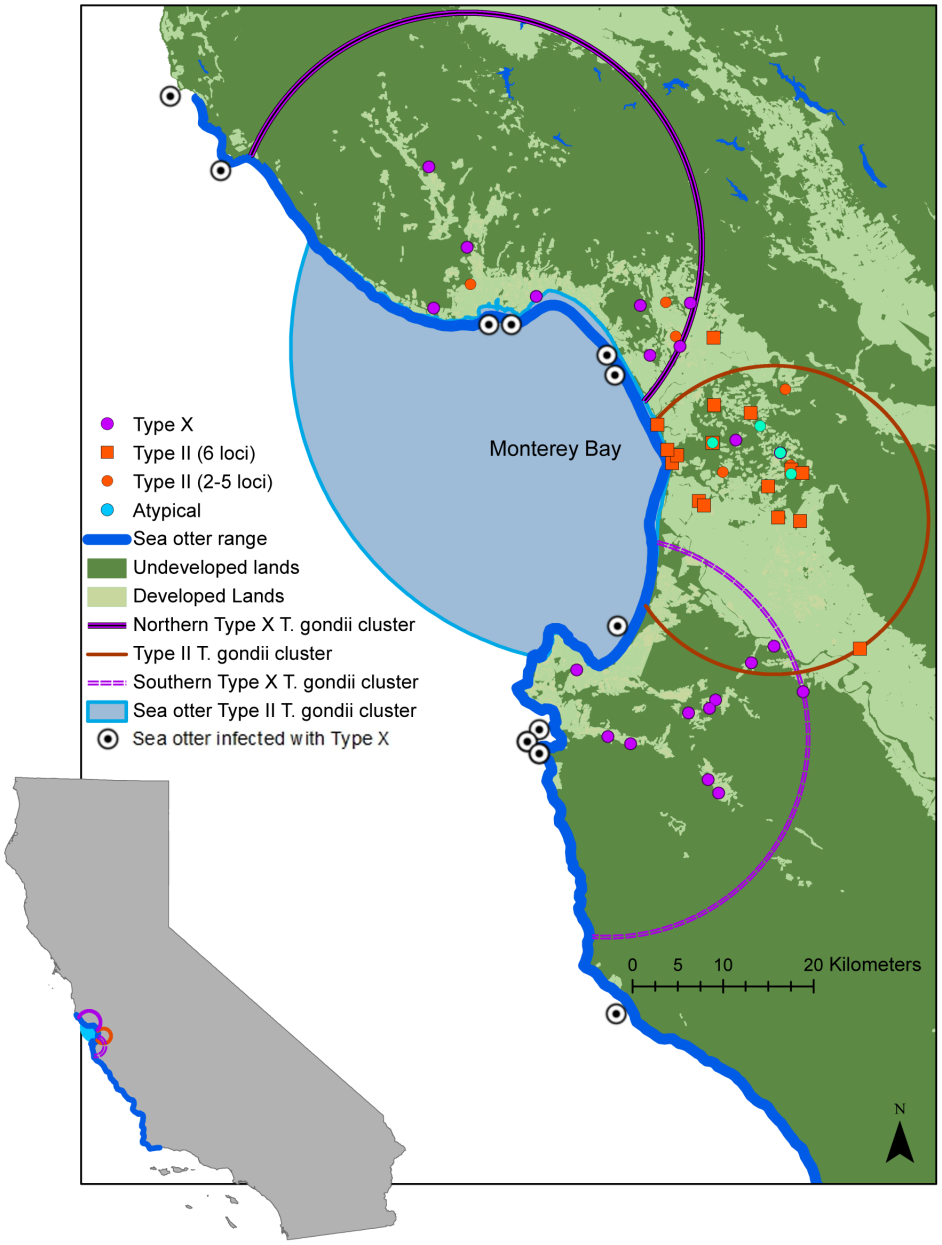
Geographic clusters of carnivores infected with *Toxoplasma gondii* strains consistent with Type II and Type X. Significant spatial clusters (P<0.05) were identified using an elliptical scanning method while adjusting for the distribution of infected wild felids, wild canids, and feral domestic cats sampled from 2006–2009. The Type X *T. gondii* clusters contained more undeveloped lands than the Type II cluster, which predominantly included developed urban, rural, and agricultural areas. The Type II cluster, which was detected using strains with Type II alleles identified at two to five loci, remained significant when a more conservative subset of strains with Type II alleles identified at six loci (B1, SAG1, SAG3, GRA6, L358, and Apico) was used. The terrestrial Type II cluster borders a previously reported cluster of Type II infection in sea otters in Monterey Bay. Previously reported Type X-infected sea otters were found at locations bordering lands in the Type X terrestrial clusters.

After adjusting for carnivore group, a significant geographic cluster of animals infected with *T. gondii* strains consistent with Type II based on alleles at two to six loci (P = 0.04) was identified near the eastern edge of Monterey Bay (Figure 3). Type II-infected animals within this cluster included feral cats and foxes. This cluster remained significant when we tested a more conservative dataset in which animals were identified as infected with Type II *T. gondii* based on alleles characterized at all six loci. The terrestrial Type II cluster borders a previously established geographic cluster of Type II *T. gondii* infection in sea otters in Monterey Bay [27]. Genotype classification in these otters was based on alleles at B1, SAG1, SAG3, and GRA6. Two geographic clusters of carnivores infected with strains consistent with Type X were detected at the northern (P<0.01) and southern (P = 0.01) ends of Monterey Bay. Type X-infected carnivores within the northern cluster included bobcats, mountain lions, foxes, and feral domestic cats. No Type X-positive feral cats were found in the southern cluster. Previously reported locations for sea otters infected with strains consistent with Type X [6], [16], [39] border the two terrestrial Type X clusters. The Type II terrestrial cluster predominantly contained developed urban, rural, and agricultural lands, while the Type X clusters included a higher proportion of undeveloped lands. The four animals with atypical strains of *T. gondii* were all sampled within 10 km of each other in an area of heavily fragmented developed and undeveloped lands bordering central Monterey Bay (Figure 3).

## Discussion

The distribution of *T. gondii* genotypes in terrestrial hosts and landscapes has important implications for parasite transmission cycles and the potential for different felids to contribute to animal and human infection in both temperate and tropical ecosystems. While Type X *T. gondii* infection has been documented in sea otters, terrestrial wildlife, and a mussel in California [16], [39], this study is the first to report infection with strains consistent with Type X in domestic cats in the coastal system, emphasizing the potential role these cats may play in environmental transmission of atypical genotypes of *T. gondii*. By analyzing strains of *T. gondii* in a large sample of geographically and temporally overlapping domestic and wild carnivores, this study also provides substantial evidence for co-existing domestic and sylvatic cycles of *T. gondii* transmission in the coastal California landscape.

### Current patterns of terrestrial and land-to-sea *T. gondii* transmission

Patterns of archetypal and atypical *T. gondii* infection derived from an opportunistic sample of feral domestic cats and wild carnivores may not perfectly reflect the true distribution of these *T. gondii* types at the population level. However, we used similar sampling approaches across species to create more accurate comparisons of *T. gondii* strains among carnivore groups. While logistical limitations prevented collection of a random sample of domestic cats and wild felids and canids, the majority of carnivores sampled (87%) were apparently healthy animals killed by vehicles or humanely euthanized in population control or depredation programs (Table S1). Eighty-five percent of the subset of carnivores with *T. gondii* DNA detected also died due to these causes. While some wild canids, 9% of the total sampled animals, were euthanized due to illness in rehabilitation centers, *T. gondii* was not identified as a cause of death for any animals in this study. Type X *T. gondii* has been associated with severe disease in California sea otters [39], but there is not any published evidence of increased virulence of this genotype in the sampled terrestrial carnivore species that would make Type X-infected animals more likely to be found dead or killed by cars than animals with archetypal infection. A strength of our approach is that domestic and wild carnivores were collected from temporally and spatially overlapping populations with animals from each group sampled in both developed and undeveloped habitats. All species were sampled in wet and dry seasons over the three year study period, with no association found between season or year of sampling and genotype of *T. gondii* infection. Given the similarity of opportunistic sampling among domestic cats, wild felids, and wild canids, it is unlikely that the collection method differentially biased the detection of archetypal and atypical genotypes among groups. Therefore, our approach allows robust comparisons of the distribution of strains consistent with Type X and archetypal genotypes among species and in the coastal landscape.

Sympatric domestic and wild felids can both shed archetypal and atypical genotype oocysts, but limited sampling of these hosts often makes their contributions to environmental parasite load difficult to define [63], [64]. Initial identification of strains consistent with Type X *T. gondii* in wild felids living along the sea otter range supported a sylvatic terrestrial source for sea otter exposure [16]. However, evidence of strains consistent with Type X infecting feral domestic cats in our study highlights their potential as a source of this novel genotype for sea otters. Using carnivore tissue samples limited our ability to amplify *T. gondii* DNA at the number of loci necessary to fully characterize Type X genotypes. However, Type X infection in sea otters and terrestrial carnivores has been classified based on RFLP and sequencing data at one (B1) to four (B1, SAG1, SAG3, GRA6) loci [16], [39], making our results comparable to previous regional studies. Detection of strains consistent with Type X in 31% of infected feral domestic cats that had *T. gondii* alleles characterized at two or more loci indicates that domestic felids can also contribute to the burden of Type X oocysts reaching the near-shore marine environment via contaminated freshwater runoff.

The distribution of genotypes in North American domestic cat populations is not fully defined, but Type X or closely related genotypes of *T. gondii* have also been identified in seven domestic cats from the southeastern United States [27]. The detection of *T. gondii* strains consistent with Type X in domestic cats in this study, but not in previous research in coastal California [16] likely reflects the larger number of domestic cats tested (n  = 168 vs. n  = 5). However, an increasing prevalence of Type X infection in feral cat populations may also have contributed to detection of Type X in these hosts. Large populations of domestic cats in the coastal California environment [54] may offset their lower prevalence of *T. gondii* Type X infection compared to wild felids, allowing these hosts to play a significant role in environmental accumulation of atypical as well as archetypal genotypes. Additionally, many domestic cats are concentrated in developed areas where conversion of natural habitats to impervious surfaces, like concrete and asphalt, facilitates pathogen transport in contaminated freshwater runoff to aquatic systems [65].

High levels of Type X *T. gondii* infection in wild felids and the predominance of archetypal genotypes in domestic cats living in the developed landscapes surrounding Monterey Bay strongly suggest that local domestic cycles driven by transmission of archetypal genotypes and independent Type X-based wild cycles co-exist in coastal central California. The proportions of archetypal and atypical genotypes, including Type X, detected in wild felids, wild canids, and domestic cats differed significantly. Mountain lions and bobcats were five to 14 times more likely to be infected with *T. gondii* strains consistent with Type X than feral domestic cats, which were predominantly infected with strains consistent with Type II. Reports of Type X in wildlife species from diverse areas of North America support a sylvatic origin and maintenance of this genotype [27].

The presence of domestic and wild *T. gondii* transmission cycles in coastal California is also supported by the differential distribution of genotypes across the coastal landscape. The majority of wild felids sampled in both developed (urban, rural, and agricultural) and undeveloped environments were infected with *T. gondii* strains consistent with Type X, whereas Type X infection in domestic cats was strongly linked to living in an undeveloped or “wild” area, where range overlap with mountain lions and bobcats was more likely. *Toxoplasma gondii* infection in foxes, which are intermediate hosts with small home ranges, offers additional insight on the genotypes present in the local environment. All foxes infected with strains consistent with Type X were sampled in undeveloped areas, whereas the majority of foxes with strains consistent with archetypal genotypes were collected from developed urban and agricultural areas near Type II-infected domestic cats. Statistically significant geographic clusters of animals infected with strains consistent with Type X were identified at the northern and southern ends of Monterey Bay, regions with predominantly undeveloped lands. In contrast, a cluster of Type II infection in the urban and agricultural lands bordering central Monterey Bay emphasized the dominance of archetypal genotypes in highly developed areas with large populations of pet and feral cats associated with humans. Emerging, severe atypical *T. gondii* infection in humans linked to wild felids in tropical jungles and archetypal strains circulating in sampled domestic animals provided evidence for the existence of similar wild and domestic transmission cycles associated with land use in French Guiana [29], [41].

The existence of domestic and wild terrestrial *T. gondii* transmission cycles in different geographic areas of the California coast has the potential to strongly impact land-to-sea transmission and sea otter infection. The Type II *T. gondii* terrestrial cluster borders the marine area where almost all of the sea otters identified as infected with Type II were sampled [39], suggesting that archetypal *T. gondii* oocysts shed by domestic cats and carried in freshwater runoff from these developed watersheds infected otters in the nearby ocean waters. Otters along the remainder of their range were predominantly infected with Type X [39], [50], and the presence of larger areas of undeveloped habitat suggested a link to Type X-infected wild felids. However, detection of *T. gondii* strains consistent with Type X in domestic cats indicates that they may also serve as a source of Type X oocysts infecting sea otters and other animals in the coastal environment, especially in areas where domestic cats share habitat with wild felids and are likely exposed to prey infected through a wild cycle of *T. gondii* transmission.

### Implications for public health and conservation

The ability of pathogens to emerge in new hosts and environments can be linked to alterations of genes and ecosystems, and changes at both levels may impact human and animal health. Although many factors can contribute to the severity of toxoplasmosis in infected animals and people, *T. gondii* genotype may play a role in virulence [32], [66]. While increased virulence of Type X in humans has not been established, other atypical genotypes have caused individual cases requiring intensive care and at least four recent and severe outbreaks of acute toxoplasmosis in humans in Canada, Brazil, Suriname, and French Guiana [40], [41], [67], [68]. Three of these outbreaks were associated with eating wild game or ingesting oocysts from environmental sources, such as contaminated water, and two were linked to wild felids. The prevalence and genotypes of human *T. gondii* infection have not been investigated in our coastal California study area. Although the prevalence of human exposure to *T. gondii* in the United States decreased from the early 1990s to the early 2000s [69], the waterborne outbreak of toxoplasmosis caused by an atypical genotype in coastal British Columbia, Canada in 1995 highlights the potential for atypical human infection to emerge in temperate environments [70]. Domestic cat infection with Type X and other atypical *T. gondii* strains therefore may increase animal and human environmental exposure to potentially more virulent strains in both temperate and tropical regions.

Domestic and wild *T. gondii* transmission cycles that overlap at the interface of developed and undeveloped lands may increase local genotype diversity or introduce existing atypical genotypes to domestic hosts with potentially severe consequences for human and animal health [29]. Evidence of both of these processes was found using fully characterized isolates from wild and anthropized areas in French Guiana, but samples from these two environments were often separated by over 50 km. As the majority of our domestic cats and wild carnivores with *T. gondii* strains identified were sampled in close proximity at the interface of developed and undeveloped lands, our results offer a complementary detailed local view of potential interactions between domestic and wild cycles of *T. gondii.* The distribution of strains consistent with Type X and Type II in the developed and undeveloped lands bordering Monterey Bay (Figure 3) provides evidence for movement of strains consistent with Type X into developed areas. Six domestic cats infected with strains consistent with Type X were sampled in highly developed urban habitats, which could indicate range and prey overlap with wild felids or emerging domestic cycles of Type X transmission. Although wild felids in California typically favor undeveloped environments [71], [72], mountain lions and bobcats sampled in urban and agricultural areas during this study and prior research [73] illustrate the potential for environmental overlap and indirect atypical *T. gondii* transmission by oocysts or infected prey to the domestic cats common in developed landscapes. Meiotic recombination following felid infection with two genotypes of *T. gondii* has the potential to generate new atypical genotypes [40]. For example, feral cat (FC 49) was infected with an atypical *T. gondii* strain characterized by an admixture of Type X and Type II alleles. This domestic cat, which lived in a developed area close to undeveloped lands, was sampled within 5 km of a Type X-infected mountain lion. The unique mixture of alleles in this strain suggests that some domestic cats, and/or their prey base, are becoming co-infected with strains with Type X and archetypal *T. gondii* alleles. The four sampled carnivores (two feral domestic cats and two foxes) infected with unique atypical strains of *T. gondii* were collected with 10 km of each other in an area with a mixture of highly fragmented developed (rural towns and agricultural areas) and undeveloped lands. A mountain lion infected with a strain consistent with Type X and domestic cats infected with strains consistent with Type II were also sampled in this area, illustrating the potential for variation in diversity of genotypes even on a small spatial scale.

Although the number of animals with atypical strains was small, their concentration in a fragmented mixture of developed and undeveloped lands could indicate that local landscape structure where domestic and wild *T. gondii* transmission cycles overlap can influence emergence of novel atypical genotypes. A mixture of fragmented developed and undeveloped lands in rural areas could increase overlap between domestic and wild cycles in several ways. Higher levels of *T. gondii* exposure are predicted in domestic cats living in rural areas due to changes in species composition of intermediate and definitive hosts as well as prey availability and domestic cat predation behavior along the urban-rural gradient [74]. Free-ranging domestic cats living in a fragmented rural landscape with increased agricultural-undeveloped edges may hunt more frequently and be more likely to contact wild felid oocysts or prey infected through the wild cycle than free-ranging domestic cats with access to cat food, human scraps, or garbage in urban areas with more discrete borders. Wild felids commonly use edge environments or riparian corridors in urban and agricultural areas, including California vineyard and orchards [75], [76]. Fragmentation could also increase mountain lion access to and predation upon livestock, potentially exposing mountain lions to domestic cycle genotypes of *T. gondii*. These factors suggest that not only an interface between developed and undeveloped lands, but also the physical structure of the interface could drive overlap between domestic and wild *T. gondii* transmission cycles.

Human development is rapidly reshaping temperate and tropical landscapes, and associated changes in pathogen transmission cycles could have significant impacts on public health and wildlife conservation [1], [2]. In many tropical areas, rapid human population growth and agricultural expansion are driving conversion of undeveloped habitats into urban, rural, and agricultural uses. In the 1980s and 1990s, the majority of new agricultural lands for crops and livestock grazing in Central and South America and Africa were converted from intact and disturbed forests [77]. Human expansion into undeveloped areas can facilitate contact among domestic cats, wild felids, and sylvatic intermediate hosts of *T. gondii*, as well as human contact with oocysts from wild felids. Higher levels of overlap between these populations have the potential to increase human exposure to atypical and possibly more virulent strains of *T. gondii* [29]. In the case of California and the threatened sea otter population, land use change may expand the range of atypical *T. gondii* genotypes in human-dominated urban and rural landscapes and simultaneously increase the movement of oocysts to the ocean. Continued development of the terrestrial landscape in temperate and tropical areas may therefore increase the number of archetypal and atypical *T. gondii* oocysts and other pathogens flowing into freshwater systems and the near-shore marine environment, where they pose a risk to sea otters, other wildlife, and humans [6], [23], [78], [79]. The molecular epidemiology approaches used to evaluate the linkages between *T. gondii* infection in domestic and wild hosts, as well as terrestrial and marine environments, have broader applications for a suite of other pathogens at the human-animal-environment interface.

## Supporting Information

Table S1 Causes of death in terrestrial carnivores sampled 2006-2009 in coastal California.
(DOCX)

Table S2 B1 locus nucleotide sequence polymorphisms for novel *Toxoplasma gondii* alleles detected in coastal terrestrial carnivores.
(DOCX)

Table S3 SAG3 locus nucleotide sequence polymorphisms for novel *Toxoplasma gondii* alleles detected in coastal terrestrial carnivores.
(DOCX)
