# Correction: Using Molecular Epidemiology to Track *Toxoplasma gondii* from Terrestrial Carnivores to Marine Hosts: Implications for Public Health and Conservation

**DOI:** 10.1371/journal.pntd.0003340

**Published:** 2014-10-27

**Authors:** The PLOS Neglected Tropical Diseases Staff

In Figure 1, the PCR negative carnivores are omitted. Please see the corrected Figure 1 here.

**Figure 1:**
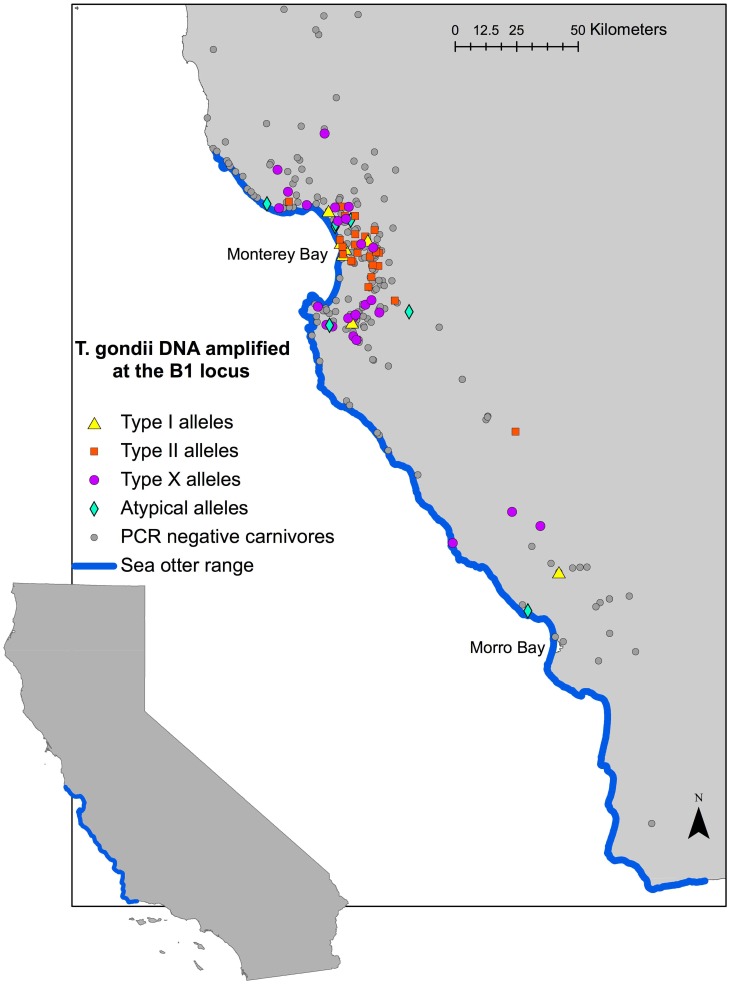
Distribution of *Toxoplasma gondii*-infected carnivores with alleles characterized at the B1 locus in coastal California. *Toxoplasma gondii* DNA was detected in 85 of 373 terrestrial carnivores (mountain lions, bobcats, foxes, coyotes, and feral domestic cats) sampled along the sea otter range in coastal California, USA, 2006–2009. Alleles present at the B1 locus were identified following nested PCR amplification of DNA extracted from tissue and parasite culture samples, restriction fragment length polymorphism (RFLP) genotyping, and direct sequencing.
