# Supplementary material for: Using Molecular Epidemiology to Track Toxoplasma gondii from Terrestrial Carnivores to Marine Hosts: Implications for Public Health and Conservation
Source: PLoS Negl Trop Dis. 2014 May 29;8(5):e2852. doi: 10.1371/journal.pntd.0002852 (PMC4038486; doi:10.1371/journal.pntd.0002852)
Supplement: Table S1 — Causes of death in terrestrial carnivores sampled 2006-2009 in coastal California. (DOCX) [file pntd.0002852.s001.docx]

**Table S1. Causes of death in terrestrial carnivores sampled 2006-2009 in coastal California.**

| Dataset | Species^a^ | N | Depredation^b^ | Shelter^c^ | Trauma | Disease | Other | Unknown |
| --- | --- | --- | --- | --- | --- | --- | --- | --- |
| Total Sampled | Feral Cat | 166 | 17 | 149 | 0 | 0 | 0 | 0 |
|  | Mtn Lion | 73 | 55 | 0 | 13 | 0 | 1 | 4 |
|  | Bobcat | 27 | 0 | 0 | 23 | 0 | 0 | 4 |
|  | Fox | 81 | 17 | 0 | 27 | 30^d^ | 1 | 6 |
|  | Coyote | 26 | 0 | 0 | 23 | 2 | 0 | 1 |
|  | **Total:** | **373** | **89 (24%)** | **149 (40%)** | **86 (23%)** | **32 (9%)** | **2 (<1%)** | **15 (4%)** |
| *T. gondii*-positive^e^ | Feral Cat | 49 | 6 | 43 | 0 | 0 | 0 | 0 |
|  | Mtn Lion | 10  11  14 | 8 | 0 | 1 | 0 | 0 | 1 |
|  | Bobcat | 11 | 0 | 0 | 9 | 0 | 0 | 2 |
|  | Fox | 14 | 3 | 0 | 1 | 9 | 0 | 1 |
|  | Coyote | 1 | 0 | 0 | 1 | 0 | 0 | 0 |
|  | **Total:** | **85** | **17 (20%)** | **43 (51%)** | **12 (14%)** | **9 (10%)** | **0** | **4 (5%)** |

^a^ Feral Cat = free-ranging, unowned domestic cats (*Felis catus*). Mtn Lion = wild felids of the species *Puma concolor*, also commonly called cougars or pumas. Fox = both red (*Vulpes vulpes*) and grey (*Urocyon cinereoargenteus*) foxes.

^b^ Animals removed through conservation programs to protect endangered shorebirds as well as mountain lions shot by wildlife wardens due to public safety concerns or predation on domestic livestock.

^c^ Animals euthanized in regional animal shelter population control programs.

^d^ One red fox had lesions consistent with severe mange, and 29 grey foxes were euthanized at wildlife rehabilitation centers during an outbreak of canine distemper.

^e^ Carnivores with *T. gondii* DNA amplified at the B1locus.
