# Supplementary material for: Using Molecular Epidemiology to Track Toxoplasma gondii from Terrestrial Carnivores to Marine Hosts: Implications for Public Health and Conservation
Source: PLoS Negl Trop Dis. 2014 May 29;8(5):e2852. doi: 10.1371/journal.pntd.0002852 (PMC4038486; doi:10.1371/journal.pntd.0002852)
Supplement: Table S2 — B1 locus nucleotide sequence polymorphisms for novel Toxoplasma gondii alleles detected in coastal terrestrial carnivores. (DOCX) [file pntd.0002852.s002.docx]

**Table S2. B1 locus nucleotide sequence polymorphisms for novel *Toxoplasma gondii* alleles detected in coastal terrestrial carnivores.**

| **B1 nucleotide position:** | **366** | **378** | **406** | **413** | **431** | **471** | **504** | **533** |
| --- | --- | --- | --- | --- | --- | --- | --- | --- |
| **Consensus sequence**^a^ | **T/C** | **G** | **A** | **C** | **T** | **T** | **G/C** | **A** |
| Type I | T | **.** | **.** | **.** | **.** | **.** | G | **.** |
| Type II | **.** | **.** | **.** | **.** | **.** | **.** | **.** | **.** |
| Type III | **.** | **.** | **.** | **.** | **.** | **.** | **.** | **.** |
| Type X | T | **.** | **.** | **.** | **.** | **.** | C | **.** |
| **RFLP allele^b^ – Animal ID** |  |  |  |  |  |  |  |  |
| Unique **_1_** – FC [39,44]^c^ | T | **.** | **.** | **.** | **.** | **.** | **.** | **.** |
| Unique **_1_** – Bobcat [10] | T | A/G | **.** | **.** | **.** | **.** | **.** | **.** |
| Unique **_1_** – Bobcat [11] | T | **.** | **.** | A/C | **.** | **.** | **.** | **.** |
| Unique **_1_** – FC [40,42] | T | **.** | **.** | **.** | **.** | **.** | **.** | A/C |
| Unique **_1_** – FC [41] | T | **.** | A/G | **.** | **.** | C/T | **.** | A/C |
| Unique **_2_** – ML [10]^d^ | C | **.** | **.** | **.** | **.** | **.** | G | **.** |
| Unique **_2_** – Fox [13] | C | **.** | **.** | **.** | **.** | **.** | G | **.** |
| Unique **_2_** – Fox [14] | C | **.** | **.** | **.** | C | **.** | G | **.** |

^a^ Consensus sequence indicates the nucleotide sequence shared by at least two of the three archetypal strains.

^b^ Subscript numbers refer to the RFLP allele identified in Table 1.

^c^ FC = free-ranging, unowned domestic cats

^d^ ML = wild felids of the species *Puma concolor*, also commonly called cougars or pumas

*Toxoplasma gondii* DNA was amplified from brain and tongue tissue samples collected from carnivores in coastal central California from 2006 through 2009 using nested PCR analyses (see Methods section).
