# Supplementary material for: Using Molecular Epidemiology to Track Toxoplasma gondii from Terrestrial Carnivores to Marine Hosts: Implications for Public Health and Conservation
Source: PLoS Negl Trop Dis. 2014 May 29;8(5):e2852. doi: 10.1371/journal.pntd.0002852 (PMC4038486; doi:10.1371/journal.pntd.0002852)
Supplement: Table S3 — SAG3 locus nucleotide sequence polymorphisms for novel Toxoplasma gondii alleles detected in coastal terrestrial carnivores. (DOCX) [file pntd.0002852.s003.docx]

**Table S3. SAG3 locus nucleotide sequence polymorphisms for novel *Toxoplasma gondii* alleles detected in coastal terrestrial carnivores.**

| **SAG3 nucleotide position:** | **981** | **1001** | **1005** | **1037** | **1044** | **1046** | **1053** | **1061** | **1076** | **1104** | **1105** |
| --- | --- | --- | --- | --- | --- | --- | --- | --- | --- | --- | --- |
| **Consensus sequence**^a^ | **C** | **G** | **A** | **T** | **G** | **G** | **C** | **C** | **A** | **G** | **G** |
| Type I | **.** | **.** | **.** | **.** | **.** | **.** | A | G | **.** | **.** | **.** |
| Type II | T | A | G | **.** | A | **.** | **.** | **.** | G | **.** | **.** |
| Type III | **.** | **.** | **.** | C | **.** | A | **.** | **.** | **.** | **.** | **.** |
| Type X | T | A | G | **.** | A | **.** | **.** | **.** | G | **.** | **.** |
| **Sequence allele^b^ – Animal ID** |  |  |  |  |  |  |  |  |  |  |  |
| II-drift **_1_** - FC [13]^c^ | T | A | G | **.** | A | **.** | **.** | **.** | G | T | **.** |
| II-drift **_2_** - Fox [5] | T | A | G | **.** | A | **.** | **.** | **.** | G | **.** | A |

^a^ Consensus sequence indicates the nucleotide sequence shared by at least two of the three archetypal strains.

^b^ Subscript numbers refer to the sequence allele identified in Table 1.

^c^ FC = free-ranging, unowned domestic cats

*Toxoplasma gondii* DNA was amplified from brain and tongue tissue samples collected from carnivores in coastal central California from 2006 through 2009 using nested PCR analyses (see Methods section).
